# Soil physicochemical properties drive the variation in soil microbial communities along a forest successional series in a degraded wetland in northeastern China

**DOI:** 10.1002/ece3.7184

**Published:** 2021-01-26

**Authors:** Xin Sui, Rongtao Zhang, Beat Frey, Libin Yang, Yingnan Liu, Hongwei Ni, Mai‐He Li

**Affiliations:** ^1^ Heilongjiang Provincial Key Laboratory of Ecological Restoration and Resource Utilization for Cold Region School of Life Sciences Heilongjiang University Harbin China; ^2^ Institution of Nature and Ecology Heilongjiang Academy of Sciences Harbin China; ^3^ Swiss Federal Research Institute WSL Birmensdorf Switzerland; ^4^ Heilongjiang Academy of Forestry Harbin China; ^5^ Key Laboratory of Geographical Processes and Ecological Security in Changbai Mountains Ministry of Education School of Geographical Sciences Northeast Normal University Changchun China; ^6^ CAS Key Laboratory of Forest Ecology and Management Institute of Applied Ecology Erguna Forest‐Steppe Ecotone Research Station Chinese Academy of Sciences Shenyang China

**Keywords:** bacterial and fungal diversity, community structure, *Deyeuxia angustifolia* wetland, high‐throughput sequencing, Sanjiang Plain

## Abstract

The Sanjiang Plain is the biggest freshwater wetland locating in northeastern China. Due to climate change and human activities, that wetland has degraded to a successional gradient from the original flooded wetland to dry shrub vegetation and a forest area with lower ground water level, which may result in changes in soil microbiologic structure and functions. The present study investigated the microbial diversity and community structure in relation to soil properties along that successional gradient. The soil physico‐chemical properties changed significantly with degradation stage. The Shannon diversity index of both soil bacteria (5.90–6.42) and fungi (1.7–4.19) varied significantly with successional stage (both *p* < .05). The community structures of soil bacteria and fungi in the early successional stages (i.e., the wetland) were significantly determined by water content, total nitrogen, and available nitrogen concentrations in soils, while those in the later successional stages (i.e., forests) were significantly structured by soil organic carbon, soil pH, and available phosphorus concentrations. These results suggest that the soil microbial structure is mainly determined by soil properties rather than by plant community such as plant species composition along successional stages.

## Graphical abstract

Soil microbial community varied during long period degrade in Sanjiang Plain of China. Variatons of soil microbe and fungi obviously changed between the wetland type and dryland type.

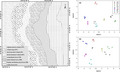

## 1 INTRODUCTION

Wetlands, accounting for 5%–8% of the Earth's surface, play a key role in regulating global greenhouse gases and act as carbon sinks (Liu, Zheng, et al., 2014; Taufik et al., 2015; Zheng et al., 2013; Wang et al., 2019). The Sanjiang Plain in northeastern China covers a region with 10.89 million hectares and features the biggest freshwater wetland with in China. Increases in human population and agricultural activities have led to a drastic decrease in the wetland area. The Sanjiang Plain consisted of fifty‐percent freshwater in 1950 (Liu & Ma, 2000), about 84% of the original wetland is now being used for agricultural purpose, especially paddy field (Liu & Ma, 2000). Moreover, due to agricultural water utilization and lower precipitation levels, the region with water as well as the water‐covered surface area has gradually declined, leading to wetlands and forest succession with degradation.

Soil microorganisms are important constituents of ecosystems and are essential for plant material decomposition (Gryta et al., 2014; Xiong et al., 2016; Wang et al., 2019) and help maintain stability of the ecosystem (Mania et al., 2014; Yin et al., 2014; Liu et al., 2019). Hence, the examination of soil microbes is critical for understanding local biogeochemical processes, emissions of greenhouse gases from soils, and degradation of pollutants (Dan et al., 2014; Song et al., 2013). Recent work has greatly targeted the structures and functions of microbial communities following the transition of wetlands to other land‐use types (Calheiros et al., 2009; Liu et al., 2008; Zhi et al., 2015; Deng et al., 2019). Studies that characterize the distribution of soil microbiota in relation to different vegetation successional stages may have practical significance (Xing et al., 2008).

Previous studies evaluated the variation in vegetation (Ji et al., 2006), greenhouse gas emissions (Song et al., 2006; Liu et al., 2010), and nutrient cycling among the atmosphere, flora, and soil (Hou et al., 2011) as a result of wetland degradation on the Sanjiang Plain. However, little research has looked into the variety as well as construction of soil bacterial as well as fungal communities in response to degradation stages.

Due to the climate change and human disturbance, the water level of Sanjiang wetland has declined and the original wetland continuously degraded, leading to forest succession surrounding the wetland. For instance, in Sanjiang Wetland Experimental Station, there are original natural wetland (NW), wetland edge (EW), shrub‐invaded wetland (IW), shrub‐dominant wetland (DW), young‐*Betula* forest (YB), mature‐*Betula* forest (MB), *Populus* and *Betula* mixed forest (PB), and conifer forest (CF) within a small distance. We used this research site to examine the impacts of degradation stages on soil microbial systems. We hypothesized that: (a) fungal community structures change more than bacterial ones from wetland to forest. In particular, based on findings from a pilot study of drying wetlands, we expected that the basidiomycota (ectomycorrhizas) would increase and the ascomycota would decrease in relative abundance; and (b) differences in soil physico‐chemical characteristics would select for distinct bacterial communities along a successional gradient from wetland to forest.

## 2 MATERIALS AND METHODS

### 2.1 Site characterization and soil selection

**FIGURE 1:**
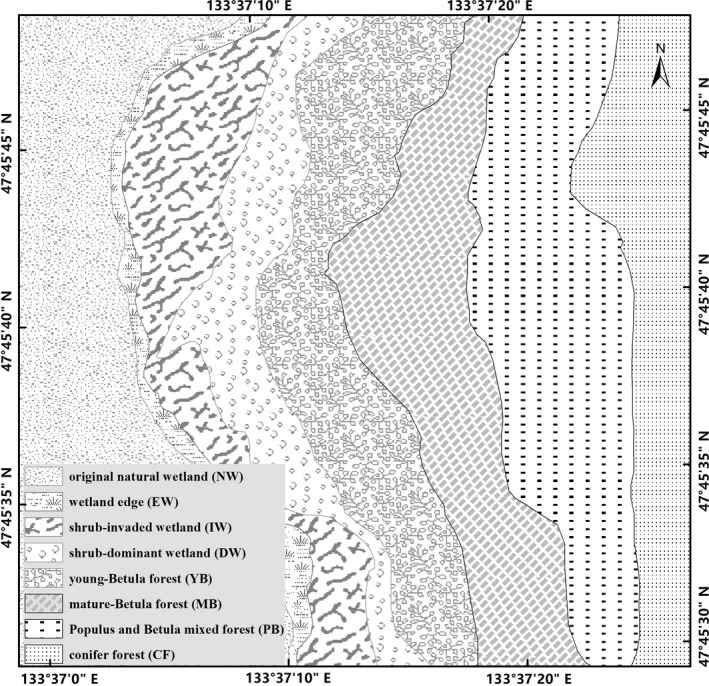
Map showing different vegetation location of the study. Note: Vegetation types: original natural wetland (NW), wetland edge (EW), shrub‐invaded wetland (IW), shrub‐dominated wetland (DW), young‐*Betula* forest (YB), mature‐*Betula* forest (MB), *Populus* and *Betula* mixed forest (PB), and conifer forest (CF)

The evaluation was performed on the Sanjiang Plain (47°35′N, 133°31′E) in northeastern China. The mean month‐long temperature spans from −21.6°C in January to 21.5°C in July, with a yearly mean temperature of 1.9°C. The average yearly precipitation is approximately 560 mm, where 80% precipitates from May to October. Eight vegetation types along a forest succession gradient in a degraded wetland were selected for this study: original natural wetland (NW), wetland edge (EW), shrub‐invaded wetland (IW), shrub‐dominant wetland (DW), young‐*Betula* forest (YB), mature‐*Betula* forest (MB), *Populus* and *Betula* mixed forest (PB), and conifer forest (CF) (Table 1, Figure 1). These eight types can be divided into two main groups, that is, an aquatic group including permanent or seasonal wetland (NW, EW, IW, and DW), and a dryland group (YB, MB, PB, and CF) (Table 1).

**TABLE 1:** Vegetation types (composition, cover, and height) along the successional gradient from flooded wetland to conifer forest

Successional stage	Abbreviation	Plant composition	Cover contribution (%)	Height (cm)	Plant Shannon diversity index
Natural wetland	NW	*Deyeuxia angustifolia*	90	105	0.44 ± 0.02e
		*Carex appendiculata*	3	70	
Wetland edge	EW	*Deyeuxia angustifolia* *Filipendula palmata* *Galium aparine* *Anemone dichotoma*	85 5 2 2	98 43 57 68	0.53 ± 0.02d
Shrub‐invaded wetland	IW	*Deyeuxia angustifolia*	55	98	0.58 ± 0.03c
		*Spiraea salicifolia*	30	112	
Shrub‐dominated wetland	DW	*Spiraea salicifolia*	50	120	0.47 ± 0.02e
		*Deyeuxia angustifolia*	30	103	
		*Anemone dichotoma*	10	65	
Young‐*Betula* forest	YB	*Betula platyphylla*	90	900	0.88 ± 0.04ab
		*Populus davidiana*	5	900	
		*Ulmus macrocarpa*	5	800	
Mature‐*Betula* forest	MB	*Betula platyphylla*	85	1,400	0.92 ± 0.03a
		*Populus sdavidiana*	5	1,400	
		*Quercus mongolica*	2	1,550	
		*Ulmu smacrocarpa*	2	1,100	
*Populus‐Betula* mixed forest	PB	*Populus davidiana*	60	800	0.85 ± 0.04b
		*Betula platyphylla*	30	850	
		*Quercus mongolica*	2	1,000	
Conifer forest	CF	*Larix gmelinii*	100	900	0.54 ± 0.03cd

Note

Plant Shannon diversity index (means ± 1 *SD*); different lowercases represent significant difference at *p* < .05 level, tested with Duncan multiple comparisons.

Three plots (10 m × 10 m) were established in each vegetation type, and the distance between any two plots was >50 m. At each plot, we identified all plant species and calculated the plant's Shannon diversity index. Soil samples (0–20 cm depth) were taken on October 15, 2016. Soil samples were collected using a sterile soil drill from 5 randomly selected locations inside each plot and were pooled to obtain a mixed soil specimen (approximately 1 kg of fresh soil) for each plot. Each soil sample was split into 2 subsamples, one of them was kept at −80°C for DNA analysis and the second one was air dried for soil physico‐chemical analyses, with soil moisture content (Mc) measured gravimetrically and soil pH quantified with a pH meter following the soil being mixed using water (1:5 w/v) for 30 min. The total organic carbon (TC) and total nitrogen (TN) concentrations were measured with an elemental analyzer (VarioEL III; Elementar Analysen systeme GmbH, Langensel bold, Germany). Soil sample was performed digestion and extraction through H_2_SO_4_‐HClO_4_, 0.5 M NaHCO_3,_ and 2.0 M KCl in succession, followed by assay with a continuous flow analytical system (SAN^++^, Skalar Analytical, the Netherlands). The catalase activity was tested using the method presented by Aebi (1984), the urease activity was established with the method detailed by Kandeler and Gerber (1988), the sucrase activity was assayed by ammonium molybdate colorimetry (Guan et al., 1984), and the acid phosphataseactivity was determined using ρ‐nitrophenyl phosphate following a method described by Eivazi and Tabatabai (1977).

### 2.2 Soil DNA extraction and PCR amplification

We removed the DNA from a 0.5‐g frozen soil specimen with a MOBIO Power Soil DNA Isolation Kit (Mo Bio Laboratories, Carlsbad, CA, USA) based on the company's directions. The extracted DNA was performed dilutionin 100 μl TE (10 mMTris–HCl, 1 mM EDTA, pH 8.0), after which it was kept at −20°C prior to employ. The DNA was quality checked and quantified using a Nano Drop ND‐1000 spectrophotometer (Thermo Scientific). We extracted the DNA of each soil sample individually and performed PCR in triplicate for each DNA sample. We amplified the V3‐V4 region of bacterial 16S rRNA (Chakravorty et al., 2007; Huse et al., 2007) and the ITS1 region of fungal ITS rRNA (Fouts et al., 2012). PCR reaction was conducted in a 25‐μl mixture incorporating every single primer with 0.5 μl (30 μM), 1.5 μl template DNA (10 ng), and 22.5 μl Platinum PCR SuperMix (Invitrogen). The PCR amplification programs for bacteria were as follows: prior to extension at 72°C for 10 min, it was placed at 95°C for 5 min, with 30 cycles at 95°C for 60 s, 55°C for 60 s, and 72°C for 60 s afterward. The programs toward fungi were as follows: prior to extension at 72°C for 10 min, it was placed at 95°C for 5 min, with 35 cycles at 95°C for 30 s, 53°C for 30 s, and 72°C for 30 s afterward. Each sample was experienced amplification in triplicate, and amplicon was removed from 2% agarose gels to purify with a Axy Prep DNA Gel Extraction Kit (Axygen Biosciences) based on the company's directions, followed by quantification with QuantiFluor‐ST (Promega). The three amplicons after purification from one DNA sample underwent pooling at equal molar concentrations as well as sequencing with paired end upon a MiSeq PE 300 platform (Illumina) based on general directions at BIONOVA Bio PharmTechnology Co., Ltd.

### 2.3 Statistical analyses

The data were tested for normality and equal variances prior to statistical analysis. Differences in soil physico‐chemical properties and enzyme characteristics among the eight vegetation types were tested with one‐way ANOVA and Duncan's tests, using SPSS 17.0 software (SPSS Inc.). Raw bacterial and fungal fastq files were performed de‐multiplexing, quality filtering, and assessing through QIIME (version 1.17). Forward and reverse reads were merged using PEAR software. Sequences with low quality with length less than 200 bp and a mean quality score less than 20 were conducted removal prior to additional analyses. Exact barcode matching was implemented, which allowed a two‐nucleotide mismatch in primer matching. Reads including unclear characters were also performed removal. The trimmed sequences were chimera checked, and those with chimeras were removed with the Uchime algorithm (Edgar et al., 2011). Just sequences having overlapping by over 10 bp were assembled based on their overlapped sequences; reads not capable of assembling were abandoned. The rest and exclusive sequences were conducted clustering at a 97% resemblance using CD‐HIT (Li, 2015) to obtain operational taxonomic units (OTUs). With respect to taxonomy, the OTUs obtained were compared to the SILVA database for bacteria as well as the UNITE database for fungi. To perform correction for differences in selection effort, we extracted the sequence of all samples to a uniform amount of data according to the lowest number of sequences for a single sample and used this data set for further community analysis. Each of the bacterial and fungal sequences has been conducted deposition in the GenBank short‐read archives with accession numbers SUB4136665 and SUB4145532, respectively.

Phylogenetic diversity (PD) of microbial community was measured according to Faith's approach, which is the sum of the total phylogenetic length of OTUs in each sample, and calculated using Picante package in R (v.3.2.5). Chao1 estimator of richness and the Shannon diversity index were determined in QIIME. Varieties in community structures (β‐diversity) were evaluated through a permutational ANOVA (PERMANOVA, number of permutations = 99,999) with the function adonis in the “vegan” package in R(version 3.2.0; R Development Core and Team, 2015) and were displayed through on‐metric multidimensional scaling (NMDS) ordination. The pair‐wise test comparison of each habitat was evaluated by multivariate permutational ANOVA (PERMANOVA). The *p*‐values from pair‐wise tests were modified for multiple comparative studies through the Benjamini–Hochberg procedure. The Mantel test was implemented to perform the estimation on the substantial effects of the environment parameters on the bacterial and fungal compositions using the vegan library in R. The H cluster of each sample (i.e., the average) was analyzed at the OTU level using R software (vegan package) established on the Bray–Curtis dissimilarity distance matrix.

We further explored the tax a responsible for the observed shifts in multivariate patterns of β‐diversity (Rime et al., 2016). Specifically, an indicator species analysis was conducted through the “labdsv” and “vegan” packages in R. Species with a high indicator value (IndVal), established on a Monte Carlo permutation with 1,000 replicates, were considered the most pivotal indicators within the community. The variations in the comparative affluence of the bacterial and fungal phyla were shown by heat map, being modeled via the vegan package in R, as previously described (Frey et al., 2016).

ANOSIM (one‐way analysis of similarities) was implemented to explore considerable variations within the CLPP of the examined vegetation varieties along a forest successional gradient in the degraded wetland (Clarke & Green, 1988). In this process, an R statistic is calculated, with *R* = 0 indicating completely random grouping, whereas *R* = 1 indicates all replicates in a group have high similarity with each other compared with any replicates crossing other groups. The global R value was utilized for the expression of the general discrepancy between the sites. A significant global R value indicates the R value differs considerably from 0, implying the compared sites are considerably disparate. Discrepancies between the sites in terms of the Bray–Curtis distance were performed testingin pair‐wise comparisons, with evaluation of the significance following the sequential Bonferroni procedure. The H cluster of each sample was analyzed at the OTU level through the vegan package in R established on the Bray–Curtis dissimilarity distance matrix.

The functional groups (guilds) of the OTUs of bacteria and fungi were inferred using FUNGuild v1.0 (Nguyen et al., 2016). The pair‐wise test comparison of each habitat was conducted using multivariate permutational ANOVA (PERMANOVA).

## 3 RESULTS

### 3.1 Soil physico‐chemical properties and soil enzymes

Except for soil acid phosphatase, all other parameters of soil physio‐chemical properties analyzed differed significantly (*p* < .05) among the eight successional stages (Table 2). Soil moisture was higher for the aquatic group than for the dryland group (Table 2). Soil pH was between 5.47 (PB) and 5.75 (NW) (Table 2), showing that dryland type had higher pH values than the wetland type. Soil total organic carbon was between 27.82 (CF) and 57.54 g/kg (DW) (Table 2), and the wetland type had higher values than the dryland type. The total nitrogen was between 2.58 (CF) and 7.62 g/kg (NW), and the wetland type had higher N content than the dryland type. Soil available nitrogen was between 165.86 (PB) and 455.25 mg/kg (NW), which was richer in wetland type than in dryland type. Soil total phosphorus was between 0.32 (YB) and 6.36 mg/kg (NW) (wetland type > dryland type). The soil available phosphorus was between 25.18 (DW) and 51.99 mg/kg (YB) (wetland type < dryland type).

**TABLE 2:** Soil physico‐chemical properties and soil enzymes in the eight vegetation types along a successional gradient in a degraded wetland

Type	Soil moisture	pH	Total organic carbon (g/kg)	Total nitrogen (g/kg)	Available nitrogen (mg/kg)	Total phosphorus (mg/kg)	Available phosphorus (mg/kg)	Catalase ml (0.1 M KM_n_O_4_)/(h.g)	Urease enzyme (mg/g)	Acid Phosphatase (mg/g)	Sucrase (mg/g)
NW	**52.79 ± 2.00a**	*5.47 ± 0.04d*	56.43 ± 3.13a	**7.62 ± 0.12a**	**455.25 ± 17.06a**	**6.36 ± 1.17a**	26.34 ± 1.05c	1.90 ± 0.02cd	36.36 ± 2.09bc	*183.44 ± 9.55a*	90.00 ± 4.37cd
EW	23.52 ± 1.63d	5.58 ± 0.08bcd	56.09 ± 2.17a	3.00 ± 0.10e	214.47 ± 28.711d	0.52±0.05c	26.45 ± 2.15c	2.04 ± 0.01b	40.92 ± 1.85ab	202.49 ± 0.80a	88.23 ± 2.06cd
IW	44.70 ± 2.07b	5.52 ± 0.03cd	38.82 ± 3.33b	5.56 ± 0.11b	381.53 ± 23.97c	0.60±0.11c	26.09 ± 2.50c	2.00 ± 0.00b	*34.23 ± 2.08c*	189.28 ± 1.91a	148.53 ± 15.18b
DW	35.62 ± 1.46c	5.69 ± 0.02ab	**57.54 ± 2.37a**	4.75 ± 0.02c	418.34 ± 10.88ac	5.18±0.19a	*25.18 ± 1.97c*	*1.86 ± 0.01d*	37.99 ± 0.45abc	192.46 ± 3.72a	106.91 ± 4.99c
YB	20.07±0.80de	*5.47 ± 0.03d*	37.88 ± 1.65b	4.28 ± 0.32d	329.55 ± 11.09b	*0.32±0.06c*	**51.99 ± 2.13a**	2.06 ± 0.03b	37.52 ± 0.30abc	200.96 ± 5.59a	148.08 ± 2.53b
MB	21.92 ± 1.83de	5.66 ± 0.02abc	36.49 ± 1.07b	7.25 ± 0.07a	437.61 ± 12.02ac	0.62±0.01c	43.15 ± 1.38b	2.02 ± 0.02b	**42.99 ± 0.14a**	184.50 ± 5.06a	119.76 ± 6.26bc
PB	*16.09±0.54e*	**5.75 ± 0.01a**	57.10 ± 2.34a	4.96 ± 0.16c	*165.86 ± 13.47d*	0.48±0.13c	49.23 ± 4.35b	**2.16 ± 0.02a**	36.39 ± 1.68bc	**214.76 ± 27.52a**	**244.84 ± 14.93a**
CF	17.18 ± 1.26e	5.58 ± 0.02bcd	*27.82 ± 2.14c*	*2.58 ± 0.17e*	189.12 ± 5.07d	2.43±0.04b	29.60 ± 1.00c	1.94 ± 0.03d	39.53 ± 0.61abc	198.30 ± 4.25a	*69.73 ± 2.00d*

Note

Statistical significance (one‐way ANOVA, *p* < .05) is indicated by different superscript letters in the same column. In each column, the largest value is shown in bold and the smallest value is shown in italics.

Vegetation types: original natural wetland (NW), wetland edge (EW), shrub‐invaded wetland (IW), shrub‐dominated wetland (DW), young‐*Betula* forest (YB), mature‐*Betula* forest (MB), *Populus* and *Betula* mixed forest (PB), and conifer forest (CF).

Except for acid phosphatase (*p* > .05), the activities of catalase, urease, and sucrase differed significantly among the eight successional stages (Table 2). The activity of catalase (ranging from 1.86 in DW to 2.16 hr/g in PB) and sucrase (ranging from 69.73 in CF to 244.84 mg/g in PB) tended to increase from wetland type to dryland type (Table 2), whereas the activity of urease (ranging from 34.23 in IW to 42.99 mg/g in MB) did not show any clear tendency (Table 2).

### 3.2 Bacterial and fungal Alpha‐ and Beta‐diversities

The Chao index and Shannon diversity index of both soil bacteria and fungi differed significantly (one‐way ANOVA, both *p* < .01) among the eight successional stages (Table 3). The bacterial Shannon diversity index was between 5.90 (CF) and 6.42 (EW), and the Chao index was between 3,360 (MB) and 4,108 (EW) (Table 3). The fungal Shannon diversity index was between 1.7 (YB) and 4.19 (DW), and the Chao index was between 458.65 (NW) and 874.17 (EW) (Table 3).

**TABLE 3:** Soil bacterial and fungal alpha‐diversity in eight vegetation types along a successional gradient in a degraded wetland in Sanjiang Plain, northeastern China

Types	Bacteria				Fungi			
	OTU	PD diversity index	Shannon diversity index	S_chao_	OTU	PD diversity index	Shannon diversity index	S_chao_
NW	3,479 ± 27.30a	**162.41 ± 1.74a**	6.1 ± 0.01b	4,044.1 ± 54c	457 ± 133.27c	203.17 ± 43.74bcd	3.4 ± 0.45bcd	*458.7 ± 81b*
EW	**3,515 ± 97.41a**	152.82 ± 1.21b	**6.4 ± 0.07a**	**4,108.5 ± 66c**	517 ± 123.35c	**291.05 ± 14.86a**	4.3 ± 0.08a	**874.2 ± 38a**
IW	3,209 ± 39.85c	152.29 ± 1.14b	6.0 ± 0.01cd	3,787.4 ± 32b	573 ± 42.39bc	212.50 ± 33.73bcd	3.5 ± 0.16bcd	599.1 ± 96ab
DW	3,302 ± 27.32b	156.21 ± 3.93b	6.1 ± 0.00bc	3,845.6 ± 35b	859 ± 62.61a	232.04 ± 15.48b	**4.2 ± 0.10ab**	594.4 ± 21ab
YB	2,899 ± 76.14ed	131.94 ± 3.44c	6.2 ± 0.03b	3,365.2 ± 75a	*428 ± 5.43c*	177.74 ± 39.77cd	*1.7 ± 0.18e*	518.3 ± 109b
MB	2,879 ± 49.57ed	130.37 ± 2.36c	6.0 ± 0.04bc	*3,360.6 ± 35a*	436 ± 131.66c	*164.07 ± 3.13d*	2.8 ± 0.05d	520.7 ± 10b
PB	2,944 ± 22.30d	130.60 ± 0.59c	*5.9 ± 0.04d*	3,540.3 ± 23a	558 ± 72.91bc	223.34 ± 15.56bc	3.3 ± 0.14cd	676.0 ± 49ab
CF	*2,832 ± 35.50*e	*128.87 ± 1.11c*	*5.9 ± 0.02d*	3,365.0 ± 26a	**725 ± 141.29ab**	247.10 ± 24.59ab	4.1 ± 0.11abc	755.0 ± 82ab

Note

Statistical significance (One‐way ANOVA, *p* < .05) is indicated by different lowercases in the same column.

In each column, the largest value is shown in bold and the smallest value in italics.

Vegetation types: original natural wetland (NW), wetland edge (EW), shrub‐invaded wetland (IW), shrub‐dominated wetland (DW), young‐*Betula* forest (YB), mature‐*Betula* forest (MB), *Populus* and *Betula* mixed forest (PB), and conifer forest (CF).

There were significant differences in the OTUs of soil bacteria among the eight successional stages (Table 3), ranging from 2,832 in CF to 3,515 in EW. The OTUs of soil fungi (428 in YB to 859 in DW) differed also significantly among the eight successional stages (Table 3).

There were significant differences in the PD diversity index of soil bacteria among the eight successional stages, ranging from 128.87 in CF to 162.41 in NW (wetland type > dryland type) (Table 3). The PD diversity index of soil fungi differed also significantly among the eight successional stages, ranging from 164.07 in MB to 291.05 in EW (Table 3), which tended to decrease from wetland type to dryland type.

**FIGURE 2:**
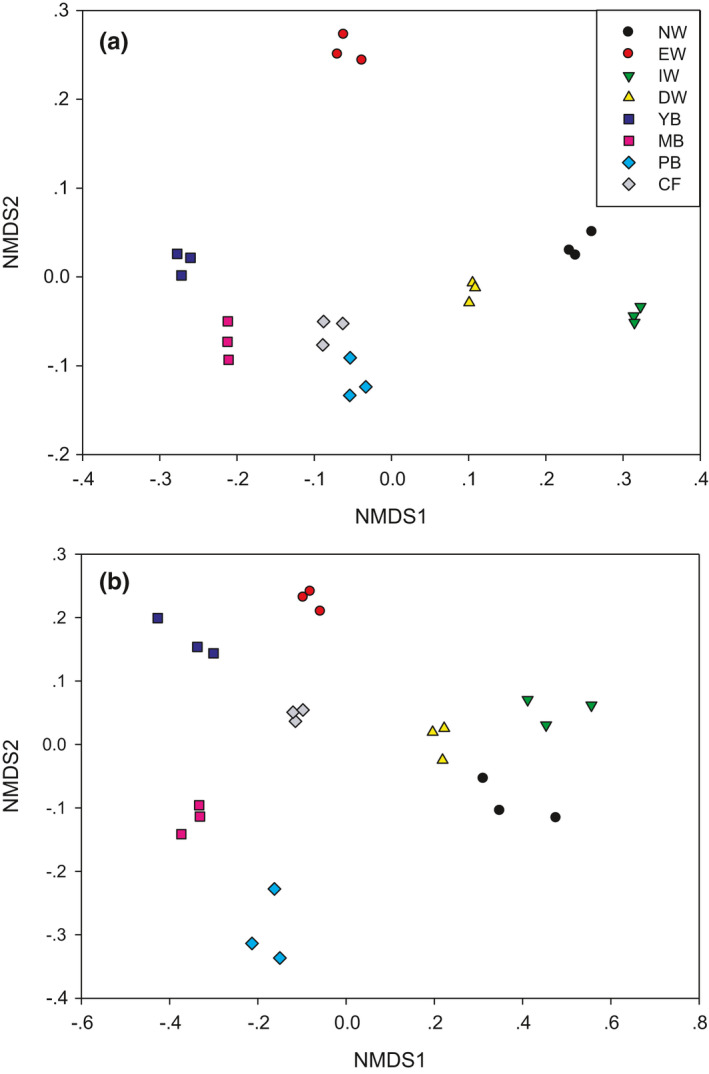
NMDS analysis of bacteria (a) and fungi (b) from the eight vegetation types along the successional gradient in a degraded wetland in Sanjiang Plain, northeastern China. Note: The β‐diversity (changes in community structures) was calculated at the OTU level (97%) based on the Bray–Curtis dissimilarity index. Vegetation types: original natural wetland (NW), wetland edge (EW), shrub‐invaded wetland (IW), shrub‐dominated wetland (DW), young‐*Betula* forest (YB), mature‐*Betula* forest (MB), *Populus* and *Betula* mixed forest (PB), and conifer forest (CF)

The NMDS separated both the bacterial and fungal community in soils of the eight successional stages into two significantly different groups, that is, an aquatic group (EW, NW, IW, and DW) and a dryland group (YB, MB, PB, and CF) (Figure 2a,b, Figures S1 and S2, Table S1).

The bacterial community of NW, DW, and IW was similar (Figure 2a and Figure S1). In the dryland type, the bacterial community of YB and MB was similar and that of PB and CF was similar with each other (Figure 2a and Figure S1). The fungal community of NW, DW, and IW was similar (Figure 2b and Figure S2). In the dryland type, the fungal community of CF and MB was similar and the fungal community of MB and PB was similar (Figure 2b and Figure S2).

### 3.3 Composition of soil fungal and bacterial communities

**FIGURE 3:**
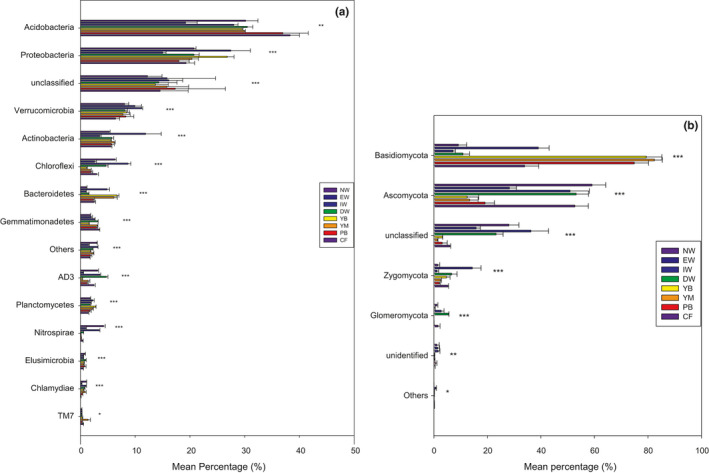
Histogram of the relative abundance of soil bacterial (a) and fungal (b) communities in eight different vegetation types along a successional gradient in a degraded wetland in Sanjiang Plain, northeastern China. Note: The level of statistical significance determined by one‐way ANOVA (***p < .001, **p < .01, *p < .05) within a particular phylum between the habitats is shown. Vegetation types: original natural wetland (NW), wetland edge (EW), shrub‐invaded wetland (IW), shrub‐dominated wetland (DW), young‐*Betula* forest (YB), mature‐*Betula* forest (YM), *Populus* and *Betula* mixed forest (PB), and conifer forest (CF)

**FIGURE 4:**
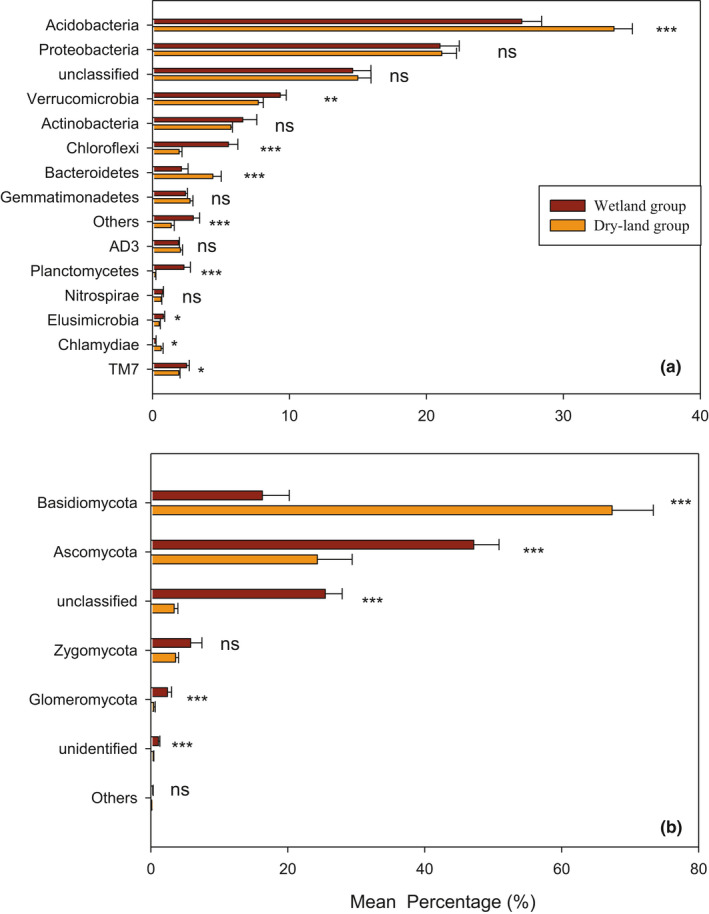
Histogram of the relative abundance of soil bacterial (a) and fungal (b) communities in wetland group (NW, EW, IW, and DW) and the dryland group (YB, YM, PB, and CF) along a successional gradient in a degraded wetland in Sanjiang Plain, northeastern China. Note: The level of statistical significance determined by one‐way ANOVA (***p < .001, **p < .01, *p < .05, ns: not significant) within a particular phylum between the habitats is shown

A total of 5,896 bacterial OTUs belonging to 15 taxonomic phyla were detected, following a decreasing order of relative abundance ofAcidobacteria > Proteobacteria>unclassified > Verrucomicrobia>Actinobacteria > Bacteroidetes>Gemmatimonadetes > Others>Planctomycetes > Chloroflexi>AD3 > TM7>Elusimicrobia > Chlamydiae>Nitrospirae (Figure 3a). All samples were overwhelmingly dominated by the phylum Acidobacteria, followed by Proteobacteria, Verrucomicrobia, and Actinobacteria. The phyla Elusimicrobia, Chlamydiae, and Nitrospirae were occasionally present at a low level of abundance (Figure 3a). Moreover, all the bacterial phyla, except unclassified species, differed significantly among the eight successional stages (*p* < .05) (Figure 3a). Acidobacteria, Proteobacteria, Verrucomicrobia, Actinobacteria, Chloroflexi, Bacteroidetes, Gemmatimonadetes, AD3, Planctomycetes, Nitrospirae, Elusimicrobia, and Chlamydiae in the aquatic group (NW, EW, IW, and DW) differed significantly from those in the dryland group (YB, MB, PB, and CF) (Figure 4a).

The relative abundance (%) of soil Acidobacteria (ranging from 19.2 in EW to 38.3 in CF) tended to increase from wetland type to dryland type (Table S2), whereas the relative abundance (%) of soil Proteobacteria (ranging from 15.0 in IW to 26.8 in YB) tended to increase from wetland type to dryland type (Table S2). The relative abundance (%) of soil Verrucomicrobia (ranging from 11.2 in IW to 6.4 in CF) tended to decrease from wetland type to dryland type (Table S2). The relative abundance (%) of soil Actinobacteria (ranging from 11.9 in EW to 3.5 in IW) did not show any clear trend (Table S2). Expect the EW and CF, the relative abundance (%) of soil Chloroflexi (ranging from 8.7 in IW to 1.1 in YB) tended to decrease from wetland type to dryland type (Table S2). Expect YB, relative abundance (%) of soil Gemmatimonadetes (ranging from 1.9 in NW to 3.4 in CF) tended to increase from wetland type to dryland type (Table S2).

A total of 3,223 fungal OTUs belonging to 6 phyla were found, following a decreasing order of relative abundance of Basidiomycota > Ascomycota>unclassified > Zygomycota > Glomeromycota > Others (Figure 3b). The phyla Basidiomycota and Ascomycota were occasionally present at low abundance (Figure 3b). Moreover, all the fungal phyla differed significantly among the eight successional stages (*p* < .05) (Figure 3b). Basidiomycota, Ascomycota, and Glomeromycota differed significantly between the dryland group and the aquatic group; Basidiomycota and Glomeromycota were more abundant in the dryland soils, and Ascomycota was more abundant in the wetlands (Figure 4b).

The relative abundance (%) of soil Basidiomycota (ranging from 7.1 in IW to 82.1 in YM) tended to increase from wetland type to dryland type (Table S3), whereas the relative abundance (%) of soil Ascomycota (ranging from 58.9 in NW to 12.4 in YB) tended to decrease from wetland type to dryland type (Table S3). Except IW and NW, the relative abundance (%) of soil Zygomycota (ranging from 14.1 in EW to 2.1 in PB) tended to decrease from wetland type to dryland type (Table S3).

### 3.4 Indicator species

**FIGURE 5:**
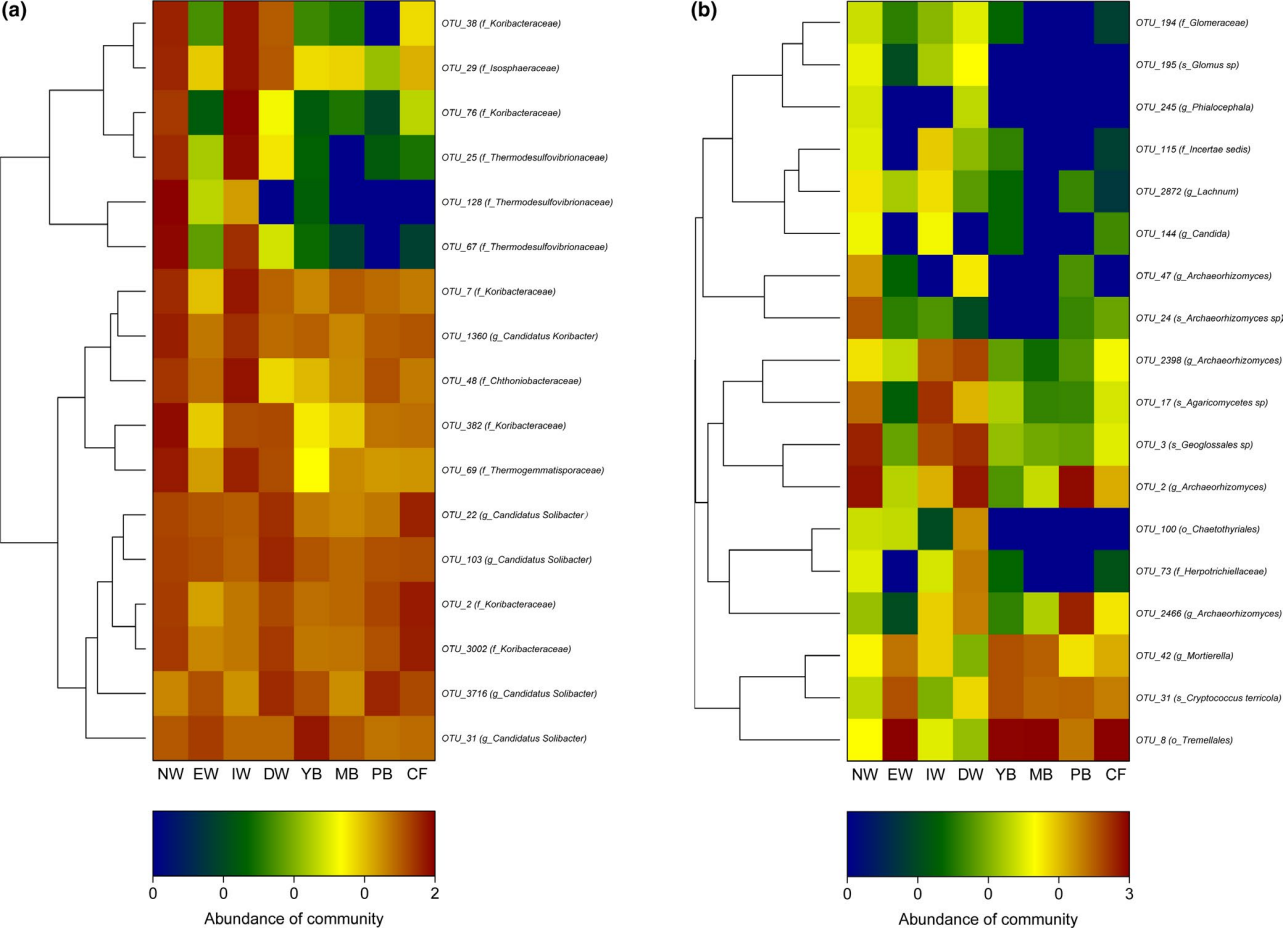
Heat maps showing changes in relative abundance of OTU. Cluster analysis based on the Ward method was performed to group samples within similar community structure and OTU with similar sample structure. The heat map represents the relative abundance of the individual OTU from low (blue) to high abundance (red). (a) represents bacteria and (b) represents fungi

The significant indicator taxa (*p* < .05) of bacteria and fungi made a response to the two habitats (aquatic and dryland group) with IndVals higher than 0.5, while the taxonomic assignment information (the highest 40 OTU numbers) is shown in Table S4 (bacteria) and S5 (fungi). For the bacteria OTUs with the highest IndVals, the taxa *Desulfovibrionaceae*, *Koribacteraceae*, *Nitrospirales*, *Koribacteraceae*, *Acidobacteria*, *Isosphaeraceae*, *Thermogemmatisporaceae*, *Chthoniobacteraceae*, *Koribacteraceae*, and *Candidatus Koribacter* were important indicator species for aquatic group, while the vital indicator taxa (including *Candidatus Solibacter*, *Koribacteraceae*) were most closely related under similar treatments in the dryland group (Figure 5a and Table S4). Regarding the fungal OTUs with the highest IndVal, the taxa *Tremellales*, *Cryptococcus terricola*, and *Mortierella* were important indicator species for aquatic group, while the vital indicator species (including *Geoglossales sp*, *Archaeorhizomyces*, *Agaricomycetes sp*, *Herpotrichiellaceae*, *Glomus sp*, *Incertae sedis*, *Glomeraceae*, *Chaetothyriales*, and *Phialocephala*) were most closely related under similar treatments in the dryland group (Figure 5b and Table S5).

### 3.5 The relationships of Soil physicochemical properties with soil bacterial and fungal communities

Mantel tests (Table S6 and S7) indicated that the soil SOC, TN, AP, and MC significantly affected the soil bacterial community across the eight stages. Moreover, TN, AN, and MC significantly affected the soil bacterial community of wetland group, and the soil pH, SOC, AN, and TP significantly affected the soil bacterial community in dryland group (Table S6).

The soil pH, TN, AN, and AP significantly affected the soil fungal community across the eight stages (Table S7). In addition, TN, AN, and TP significantly affected the fungal community in wetland group, and the soil pH, SOC, and AP significantly affected the fungal community in dryland type.

### 3.6 Functional group of fungal community

**FIGURE 6:**
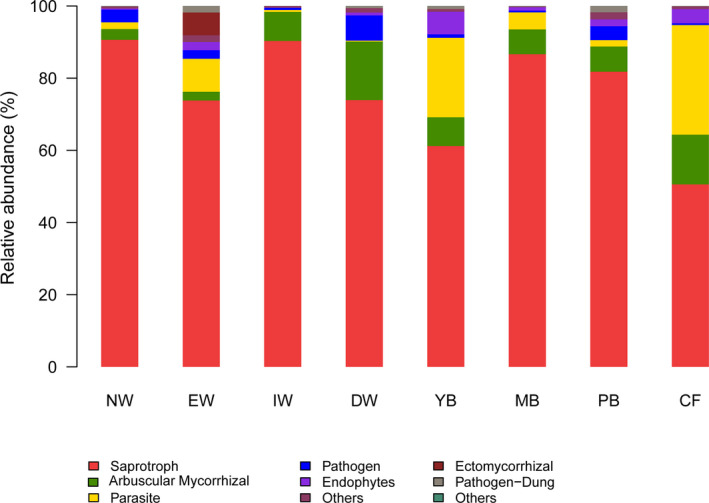
Compositions of fungal guilds inferred by FUNGuild. Note: Vegetation types: original natural wetland (NW), wetland edge (EW), shrub‐invaded wetland (IW), shrub‐dominated wetland (DW), young‐*Betula* forest (YB), mature‐*Betula* forest (YM), *Populus* and *Betula* mixed forest (PB), and conifer forest (CF)

The functional groups (guilds) of fungi differed significantly among the eight successional stages (*F* = 42.94, *p* < .001), and between the aquatic group and dryland group (*F* = 4.79, *p* < .001) revealed by Adonis analysis (Table 4), indicating that the fungal guilds change significantly with wetland degradation. The relative abundance of fungal functional groups varied with successional stage (Figure 6). The dominant groups of fungi were saprotrophs, arbuscular, and ectomycorrhizal mycorrhizal fungi, parasites, pathogens, and endophytes across the eight stages. Saprotrophs, mycorrhizal, parasite, and pathogen were the dominant functional groups in the wetland group, possibly because the wetland habitat has less oxygen.

**TABLE 4:** Adonis analysis of fungal guilds to compare the difference among the eight vegetation types, and between the wetland type and dryland type along the successional stages in a degraded wetland in Sanjiang Plain, northeastern China

Characteristic	Sum of Sqs	Mean Sqs	*F*	*R* ^2^	*p*
Habitat	8.45	1.21	42.94	**0.95**	.001
Wetland group versus Dryland group	1.59	1.59	4.79	**0.18**	.001

## 4 DISCUSSION

### 4.1 Soil bacterial and fungal community in relation to wetland degradation

The predominant phyla in the soil bacterial and fungal communities of Sanjiang Plain were generally consistent across the eight successional stages, but there were differences in their relative abundances (Figure 3). The observed predominance of Proteobacteria and Acidobacteria was not out of expectation due to the descriptions of these groups as general inhabitants for wetland and forest soils (Li et al., 2016; Liu, Zheng, et al., 2014; Zhang et al., 2014). Actinobacteria, as featured as substantial inhabitants of soils (Fierer et al., 2012; Wang et al., 2014), but were present at a low abundance in our study, possibly because Actinobacteria are sensitive to acidity and are more common in soils with neutral and alkaline pH (Joos et al., 2001; Klopatek et al., 1988). Soils are acidic at our study site, potentially affecting the Actinobacterial populations growth.

The observed predominance of Basidiomycota and Ascomycota was not out of expectation due to the descriptions for these groups as general inhabitants for wetland and forest soils (Han et al., 2018; Sui, 2017). In addition, Ascomycota was more frequently identified in aquatic group than dryland group, while the Basidiomycota was more frequently identified in dryland group than aquatic group. The wetland soils had the highest amount of organic matter in comparison with the forest soil varieties (Table 2). Consequently, unclassified members occupy a comparatively high proportion of the wetland soil. Moreover, the phyla Zygomycota and Glomeromycota were primarily detected within aquatic group, while Basidiomycota predominated in dryland group.

Ascomycota and Basidiomycota are the main decomposers in the soil (Yelle et al., 2010). Most Ascomycota are saprophytic fungi that can decompose recalcitrant organic material, such as lignin and keratin, which play an important role in nutrient cycling (Christin et al., 2014). In addition, some fungi are more sensitive to vegetation types than bacteria, such as mycorrhizal fungi that are symbiotic with plants (Bardgett & McAlister, 1999), and Basidiomycota that degrade plant residues lignin (Bossuyt et al., 2001). Basidiomycota may have poor adaptability to wetland soil environment (aquatic group), so the relative abundance of Basidiomycota is less than that of forest types (dryland group).

Fungal communities are strongly influenced by vegetation type (different litter quality), resulting in significant differences in soil physical as well as chemical characteristics, including soil pH, water content, biomass and chemical properties of litter, and belowground carbon concentration (Raich et al., 2014). Previous studies suggested that plants may have an impact on the soil microbial community structure via affecting the soil environment (see also Tables 1 and 2). Soil microbial diversity has a positive correlation with plant community diversity (Zhang et al., 2010). The impact on the fungal community occurs through the rhizosphere secretions by the plants because different plants produce different secretions that affect the soil microbial community constitutions (Nayyar et al., 2010); on the other hand, plants form a mutualistic symbiosis with mycorrhizal fungi. This interspecies cooperation enhances the competitiveness of cooperative soil fungi, thus inhibiting the life activities of other fungal species in a limited resource, thus affecting soil fungal community structure (Fang & Tolgor, 2014).

We found that Ascomycota is the dominant phylum of the Sanjiang Plain wetland, consistent with the conclusion that Ascomycota was found to be the dominant gate in freshwater (Vijaykrishna et al., 2006). The water supply of Sanjiang wetland mainly depends on rainfall, rivers, and melting snow, which is a typical freshwater seasonal marsh wetland, so Ascomycota becomes the dominant bacteria. The rhizosphere effects of plants have a dominant effect on the diversity of fungal communities, even on the same ecological environment (Costa et al., 2010; Yang et al., 2017) reported that in the soil of the Loess Plateau, due to the restoration of vegetation, the community dominated by Basidiomycota gradually transitioned to Ascomycota.

The aquatic group was dominated by saprotrophs, mycorrhizal, parasite, pathogen, possibly because the wetland habitat less oxygen. Moreover, Yang et al. (2018) found that the arbuscular mycorrhizal fungi (AMF) were obviously lower than agriculture land, so we inferred that low oxygen in wetland soil to a certain extent limits the symbiosis of AMF and plants. Thus, it was generally accepted that AMF played no or a limited role only in wetland ecosystems; however, in recent years, many studies have demonstrated the importance of the symbiosis of AMF with plants in wetlands (Carvalho et al., 2004; Dunham et al., 2003). Our previous study indicated a high amount of AMF in the *Deyeuxia angustifolia* wetland of Sanjiang plain (Yang et al., 2018).

The structure and composition of bacterial and fungal communities differed markedly across the eight successional stages (Figure 3), which confirms that vegetation characteristics can lead the presence of different soil bacteria and fungi since the diverse microhabitats is formed to lay foundation for distinct species gathering (Bell et al., 2009; Zak et al., 2003). Bacterial abundance and diversity were highest in EW, and fungal abundance and diversity were highest in DW and EW, respectively, indicating that vegetation type had an effect on edaphic ecology. Similarly, Lienhard and Sébastien (2014) found that maximum bacterial and fungal diversity occurred under different utilization schemes. The existence of distinct bacterial and fungal communities in the various vegetation varieties was in all likelihood as a consequence of many parameters, for example, soil characteristics, plant community, and microclimate (Fu et al., 2006; Zhang et al., 2013). Higher plant diversity has been suggested to have a positive correlation with bacterial and fungal variety (Zul et al., 2007). Compared with the forest types, wetland TOC, AN, and AP were considerably higher (Table 2). Aboveground vegetation affects soil physico‐chemical characteristics including the pH, soil organic matter, soil structure, and microclimate (An et al., 2018). Changes of these parameters can have an impact on the physical as well as metabolic niche variety in soils, leading to various constitutions of microbial communities (Hartmann et al., 2017).

### 4.2 Relations between soil bacterial and fungal structures as well as soil physicochemical properties

A recent investigation showed that a high microbial phospholipid fatty acid content is normal within soils with high C as well as N contents, providing adequate nutrient resources toward microorganisms (Zelles & Bai, 1994). The high bacterial community diversity in the wetlands in our study may have resulted from the SOC, TN, AP, and MC concentrations (Table S6), and that of the fungal community may have resulted from the concentrations of pH, TN, AN, and AP (Table S7). Bacterial and fungal compositions are ruled by the discharge of plant root exudates. Lynch and Whipps (1990) found that up till 40% plant dry matter is discharged to the soil being exudates. In addition, the bacterial and fungal community constitution among the eight vegetation varieties may have been driven through other biotic and/or abiotic factors associated with the soil quality and the growth of aboveground vegetation.

Moreover, soil pH can have an significant impact on microbial communities (Grayston et al., 2001). In our study, the Mantel tests exhibited that pH was a very crucial environment parameter influencing soil microorganisms (Tables S6 and S7). The low pH stress has a considerable impact on the general variety as well as constitution of microbial communities across various terrestrial environments (Fierer & Jackson, 2006). Meharg and Killham (1990) documented that a comparatively high pH value favored bacterial growth, both in a direct and indirect way, since a high pH increased exudation from plant species within grasslands.

Changes of soil pH during wetland succession also have a significant effect on the microbial community structure. Soil pH, as documented, can affect the microbial community spatial distribution. The composition, diversity, and dominance of bacteria in black soil in northeastern China could varied through soil pH and soil organic carbon (Liu, Sui, et al., 2014). Furthermore, the spatial distribution of microbes can be affected by soil pH (Shen et al., 2013). During the process of wetland transformation from the original wetland to the meadow wetland at our study site, the soil pH increased from 5.70 to 7.43, a change that was associated with an increase in microbial diversity, possibly because most of the bacteria are sensitive to acidic conditions and the increase in pH is beneficial for the growth and reproduction of some microorganisms. Many studies have shown that pH is frequently correlated with the bacterial community structure in a variety of ecosystems (Clegg, 2006; Singh et al., 2008). There are two potential reasons for this phenomenon: (a) microorganisms in the soil environment have unique optimal pH values, so a change in pH has a large impact on the microbial community diversity (Rousk et al., 2010), and (b) pH can have an impact on the microbial community in a round about way via affecting the soil nutrient supply and the quantity of root exudates from various plant species (Ren et al., 2018).

The dominant factors affecting the soil microbial community diversity have relation with the soil's physical as well as chemical characteristics, and the vegetation composition status or successional stages (Lamb et al., 2011). Wetland degradation leads to differences in vegetation type, which in turn cause changes in soil properties and aboveground communities. We found that the original natural wetland (NW) and wetland edge (EW) both had a high bacterial Shannon diversity index, meaning that a large number and even distribution of bacterial species existed in these vegetation types. However, the fungal Shannon diversity index was low in the original natural wetland (NW) and high in the wetland edge (EW) and coniferous forest (CF). Hence, the variation in bacterial diversity along the wetland degradation gradient differed from that of fungi. This may have occurred because the soil environment of the original natural wetland (which included a large amount of litter and a high soil moisture content) was more suitable for the survival of bacteria. (Han et al., 2003) found more soil bacterial taxa in natural restored grassland than in a shrub environment. (Liu et al., 2014c) reported that herbaceous vegetation changed the soil microenvironment, which led to variations within the soil microbial community structure. In addition, Xiang et al. (2008) found that a higher soil moisture content can increase soil bacterial activity. Hence, a change in the soil physico‐chemical properties affects the microenvironment of bacteria, which is the key factor that affects the distribution, diversity, and activity of bacterial communities.

The soil organic carbon and total nitrogen concentrations within the original natural wetland (NW) were higher than those in the degraded wetland vegetation types (Table 2), indicating that NW was more suitable for soil microbial metabolism and growth. The Mantel analysis on the soil microbial community structure at different wetland degradation stages showed that soil physical as well as chemical properties had different influences on the structure of bacteria and fungi (see Tables S6 and S7). The Mantel test reveals that alterations within the soil fungal and bacterial communities were dependent heavily on the soil properties within every single forest and wetland type (Tables S6 and S7). For the aquatic group, the soil MC was the leading influencing factor that determined the soil bacterial community, along with the non‐negligible effects imposed by the soil TN and AN content. For the dryland group, the soil AN content was the leading influencing factor that determined the soil bacterial community, along with important effect brought by the SOC, pH, and TP. For the aquatic group, the soil MC was the leading influencing factor that determined the soil fungal community, along with considerable influence brought by soil TN, AN, and TP contents. For the dryland group, the soil pH was the leading influencing factor that determined the soil fungal community, along with considerable influence imposed by SOC and AP.

Many studies have shown that soil physical as well as chemical properties affect soil bacterial as well as fungal community structures. For instance, Degens et al., (2000) documented that SOC is an important factor that maintains the diversity of a microbial community. The nitrogen content has also been reported to affect soil microbial community diversity. For example, an increase in the soil nitrogen content can increase the soil microbial growth rate and quantity, and the effective cycling of nitrogen plays a major role in sustaining ecosystem functions (He et al., 2009). Further, different seasons lead to a substantial effect on soil microbial community composition as well as activity (He et al., 2009). There is currently a lack of close monitoring of different successional stages of vegetation, and the seasonal dynamics of soil microbes should be considered in more detail in future studies.

## 5 CONCLUSIONS

We observed differences in the soil properties and the characteristics of the soil bacterial as well as fungal communities among the eight successional stages along a wetland degradation. The bacterial and fungal Shannon diversity index varied considerably among the eight stages. The bacterial and fungal community structures showed similar distinctions between aquatic and dryland group. Our data suggest that soil pH and resource availability (soil organic carbon, available nitrogen, total nitrogen, and total phosphorus concentrations) are the primary drivers of soil fungal and bacterial community composition along the wetland degradation gradient. This study improved our insight on soil microbial structure as well as function in a typical wetland ecosystem, and the results can be used to predict the impact of future global changes on wetland ecosystems.

## CONFLICT OF INTEREST

None declared.

## AUTHOR CONTRIBUTIONS

**Xin Sui:** Conceptualization (lead); data curation (lead); investigation (lead); methodology (lead); validation (lead); visualization (lead); writing–original draft (lead); writing–review and editing (lead). **Rongtao Zhang:** Data curation (supporting); formal analysis (supporting); resources (supporting); supervision (supporting). **Beat Frey:** Writing‐original draft (supporting); writing–review and editing (supporting). **Libin Yang:** Investigation (supporting); writing–review and editing (supporting). **Yingnan Liu:** Formal analysis (supporting); funding acquisition (supporting). **Hongwei Ni:** Project administration (lead); resources (lead). **Maihe Li:** Data curation (supporting); formal analysis (supporting); writing–original draft (supporting); writing–review and editing (supporting).

## Supporting information

Figure S1

Figure S2

Table S1–S7

## Data Availability

Raw data of bacterial and fungal sequences have been deposited into the NCBI Sequence Read Archive under accession number SUB4136665 and SUB4145532, respectively. https://doi.org/10.22541/au.158333878.83191028
