# Supplementary figures and images for: Soil physicochemical properties drive the variation in soil microbial communities along a forest successional series in a degraded wetland in northeastern China
Source: Ecol Evol. 2021 Jan 26;11(5):2194–208. doi: 10.1002/ece3.7184 (PMC7920768; doi:10.1002/ece3.7184)

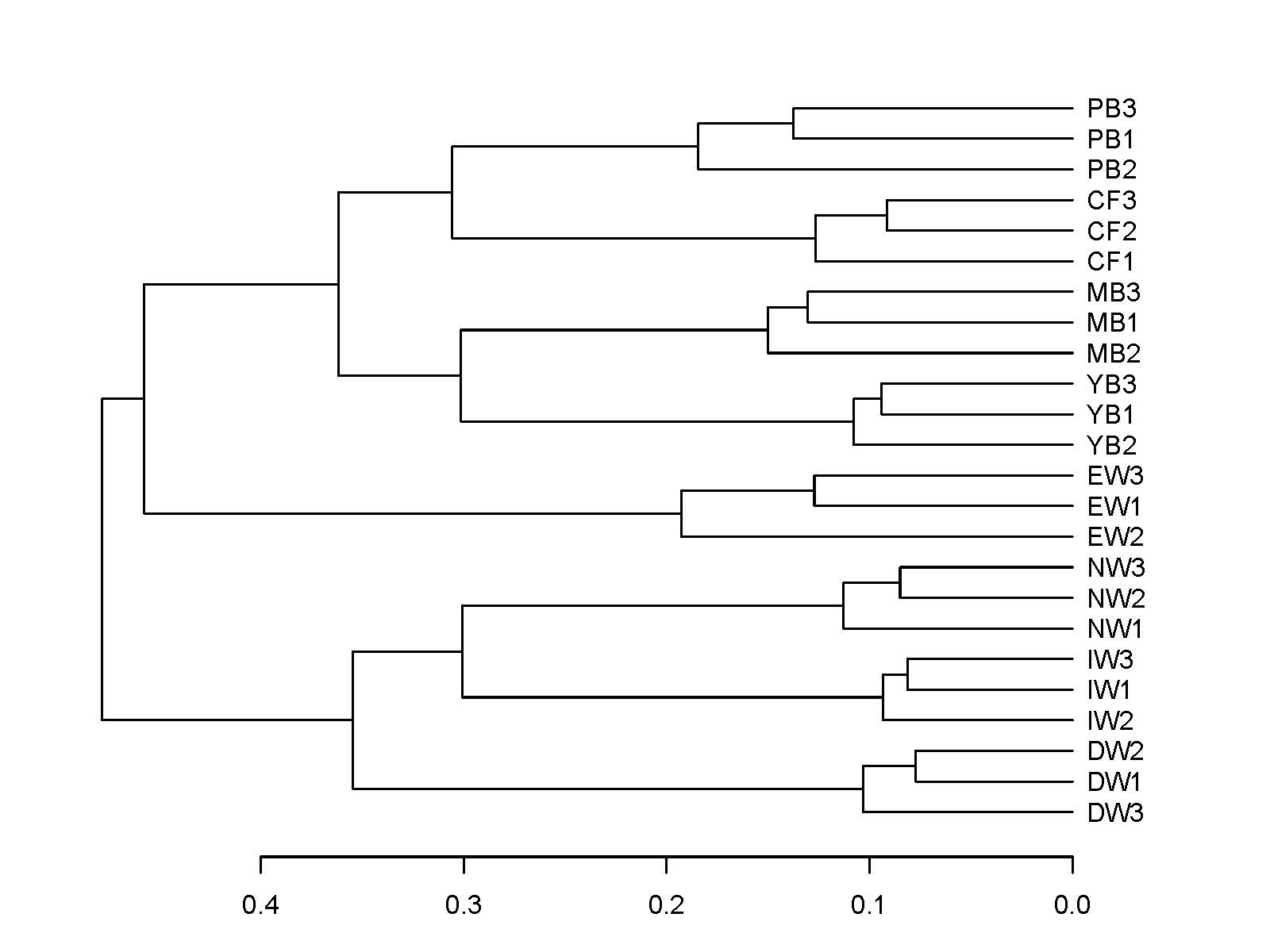

Supplement: Supplementary file 1 — Figure S1 [file ECE3-11-2194-s001.jpg]

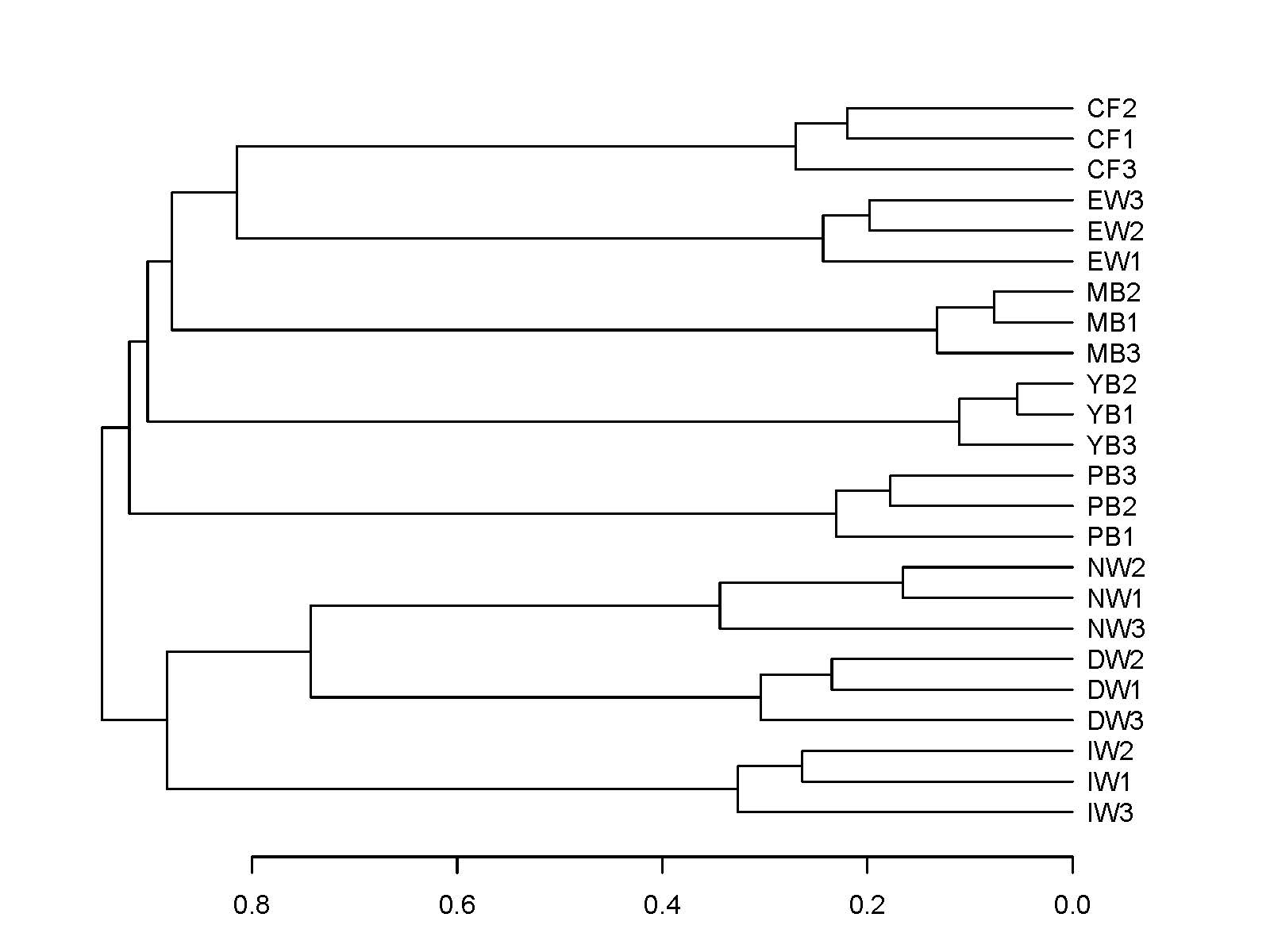

Supplement: Supplementary file 2 — Figure S2 [file ECE3-11-2194-s002.jpg]
