# Supplementary material for: Soil physicochemical properties drive the variation in soil microbial communities along a forest successional series in a degraded wetland in northeastern China
Source: Ecol Evol. 2021 Jan 26;11(5):2194–208. doi: 10.1002/ece3.7184 (PMC7920768; doi:10.1002/ece3.7184)
Supplement: Supplementary file 3 — Table S1–S7 [file ECE3-11-2194-s003.doc]

Table S1 Adonis analysis of bacteria and fungi comparing two types along the successional stages in a degraded wetland in Sanjiang Plain, northeastern China.

|  | Bacteria | | Fungi | |
| --- | --- | --- | --- | --- |
|  | R^2^ | *P* | R^2^ | *P* |
| Wetland versus dry group | **0.34** | 0.001 | **0.19** | 0.001 |

Wetland included original natural wetland (NW), wetland edge (EW), shrub-invaded wetland (IW), shrub-dominated wetland (DW); Dryland included young-*Betula* forest (YB), mature-*Betula* forest (MB), *Populus* and *Betula* mixed forest (PB), and conifer forest (CF).

Table S2 The relative abundance (>1%) of soil bacterial community in eight different vegetation types along a successional gradient in a degraded wetland in Sanjiang Plain, northeastern China.

| Samples | Acidobacteria(%) | Proteobacteria(%) | Verrucomicrobia(%) | Actinobacteria(%) | Chloroflexi(%) |
| --- | --- | --- | --- | --- | --- |
| NE | 30.2±2.2b | 20.7±0.4b | 8.1±0.7cd | 5.3±0.2bc | 6.3±0.2b |
| EW | 19.2±2.1c | 27.5±3.6a | 10.0±1.2ab | 11.9±2.9a | 2.6±0.3d |
| IW | 28.0±0.8b | 15.0±0.6c | 11.2±0.2a | 3.5±0.3c | 8.7±0.5a |
| DW | 30.5±1.0b | 20.7±1.0b | 8.1±0.5cd | 5.7±0.3b | 4.7±0.4c |
| YB | 29.5±0.2b | 26.8±1.2a | 8.5±0.5bc | 5.5±0.2b | 1.1±0.2f |
| YM | 30.0±0.1b | 20.4±1.2b | 7.8±1.3cd | 6.2±0.2b | 1.8±0.2e |
| PB | 37.0±4.6a | 18.0±1.9b | 8.3±1.4bc | 5.7±0.5b | 1.9±0.3e |
| CF | 38.3±1.7a | 19.3±1.6b | 6.4±0.8d | 5.6±0.2b | 3.0±0.3d |
| Samples | Bacteroidetes(%) | Gemmatimonadetes(%) | Planctomycetes(%) |  |  |
| NE | 1.1±0.1e | 1.9±0.0d | 1.8±0.1c |  |  |
| EW | 4.9±0.4c | 2.0±0.2d | 2.3±0.3b |  |  |
| IW | 1.0±0.1e | 2.6±0.1c | 1.8±0.2c |  |  |
| DW | 1.5±0.0e | 3.2±0.1b | 1.7±0.1c |  |  |
| YB | 6.7±0.4a | 1.6±0.1e | 2.8±0.1a |  |  |
| YM | 6.1±0.6b | 3.1±0.1b | 2.2±0.1b |  |  |
| PB | 2.4±0.2d | 2.9±0.2b | 1.7±0.2c |  |  |
| CF | 2.5±0.3d | 3.4±0.1a | 1.4±0.1d |  |  |

Table S3 The relative abundance (>1%) of soil fungal community in eight different vegetation types along a successional gradient in a degraded wetland in Sanjiang Plain, northeastern China.

| Samples | Basidiomycota(%) | Ascomycota(%) | Zygomycota(%) |
| --- | --- | --- | --- |
| NE | 9.0±3.1d | 58.0±6.0a | 1.4±0.7d |
| EW | 38.4±4.0c | 27.8±2.6b | 14.1±3.2a |
| IW | 7.1±0.9d | 50.6±7.1a | 1.2±0.7d |
| DW | 10.7±2.4d | 52.2±4.5a | 6.5±2.0b |
| YB | 78.9±5.5ab | 12.4±4.3c | 4.7±1.4bc |
| YM | 82.1±3.0a | 13.3±3.2c | 2.5±0.3cd |
| PB | 74.7±5.4b | 19.1±3.5c | 2.1±0.4cd |
| CF | 33.8±5.3c | 52.5±5.0a | 5.2±0.3b |

Table S4 Indicator taxa of bacteria of two groups along the successional stages in a degraded wetland in Sanjiang plain, northeast of China (IndVal > 0.5, relative abundance > 1%).

| Group | Indicator species | indval | P value | Taxonomy |
| --- | --- | --- | --- | --- |
| Wetland group | OTU_67 | 0.99 | 0.001 | *f_**Thermodesulfovibrionaceae* |
|  | OTU_128 | 0.99 | 0.001 | *f_Thermodesulfovibrionaceae* |
|  | OTU_76 | 0.99 | 0.001 | *f__**Koribacteraceae* |
|  | OTU_25 | 0.98 | 0.002 | *f_Thermodesulfovibrionaceae* |
|  | OTU_87 | 0.97 | 0.001 | *o_**Nitrospirales* |
|  | OTU_38 | 0.89 | 0.001 | *f_**Koribacteraceae* |
|  | OTU_81 | 0.87 | 0.001 | *p_**Acidobacteria* |
|  | OTU_29 | 0.80 | 0.001 | *f_**Isosphaeraceae* |
|  | OTU_69 | 0.72 | 0.001 | *f_**Thermogemmatisporaceae* |
|  | OTU_382 | 0.69 | 0.001 | *f__Koribacteraceae* |
|  | OTU_48 | 0.66 | 0.001 | *f_**Chthoniobacteraceae* |
|  | OTU_7 | 0.65 | 0.001 | *f_**Koribacteraceae* |
|  | OTU_32 | 0.60 | 0.002 | *p_AD3* |
|  | OTU_33 | 0.57 | 0.004 | *c_Alphaproteobacteria* |
|  | OTU_1360 | 0.56 | 0.001 | *g_**Candidatus Koribacter* |
|  | OTU_50 | 0.54 | 0.003 | *c_Betaproteobacteria* |
| Dry land group | OTU_5 | 0.51 | 0.001 | *c_Gemmatimonadetes* |
|  | OTU_10 | 0.50 | 0.009 | *p_AD3* |
|  | OTU_26 | 0.48 | 0.069 | *p_Chloroflexi* |
|  | OTU_2 | 0.47 | 0.019 | *f_Koribacteraceae* |
|  | OTU_3716 | 0.47 | 0.019 | *g_**Candidatus Solibacter* |
|  | OTU_3002 | 0.45 | 0.043 | *f_**Koribacteraceae* |
|  | OTU_55 | 0.45 | 0.041 | *o_Solibacterales* |
|  | OTU_22 | 0.39 | 0.339 | *g_Candidatus Solibacter* |
|  | OTU_144 | 0.48 | 0.045 | *g_Candida* |
|  | OTU_24 | 0.36 | 0.111 | *g_Archaeorhizomyces* |
|  | OTU_3 | 0.37 | 0.728 | *p_Acidobacteria* |
|  | OTU_103 | 0.36 | 0.689 | *g_Candidatus Solibacter* |
|  | OTU_31 | 0.53 | 0.001 | *g_Candidatus Solibacter* |

IndVal > 0.5 were selected for analyze. P.values are based on 1000 Monte Carlo permutations.

Taxonomy of OTUs were identified by using blastn in SILVA: p, phylum; c, class; o, order; f, family; g, genus; s, species.

Table S5 Indicator species of fungi of eight vegetation types along the successional stages in a degraded wetland in Sanjiang plain, northeast of China (IndVal > 0.5, relative abundance > 1%).

| Group | Indicator  species | IndVal | P value | Taxonomy |
| --- | --- | --- | --- | --- |
| Wetland group | OTU_8 | 0.95 | 0.001 | *o_**Tremellales* |
|  | OTU_31 | 0.80 | 0.001 | *s_**Cryptococcus terricola* |
|  | OTU_42 | 0.73 | 0.002 | *g_**Mortierella* |
| Dry land group | OTU_3 | 1.00 | 0.001 | *s_**Geoglossales sp* |
|  | OTU_2 | 0.98 | 0.001 | *g_**Archaeorhizomyces* |
|  | OTU_2398 | 0.97 | 0.001 | *g_Archaeorhizomyces* |
|  | OTU_2466 | 0.93 | 0.002 | *g_Archaeorhizomyces* |
|  | OTU_17 | 0.91 | 0.001 | *s_**Agaricomycetes sp* |
|  | OTU_2872 | 0.82 | 0.007 | *g_Lachnum* |
|  | OTU_73 | 0.75 | 0.001 | *f_**Herpotrichiellaceae* |
|  | OTU_195 | 0.67 | 0.002 | *s_**Glomus sp* |
|  | OTU_115 | 0.66 | 0.003 | *f_**Incertae sedis* |
|  | OTU_194 | 0.66 | 0.003 | *f_**Glomeraceae* |
|  | OTU_47 | 0.58 | 0.003 | *g_Archaeorhizomyces* |
|  | OTU_100 | 0.58 | 0.01 | *o_**Chaetothyriales* |
|  | OTU_245 | 0.50 | 0.01 | *g_**Phialocephala* |

IndVal > 0.5 were selected for analyze. P.values are based on 1000 Monte Carlo permutations.

Taxonomy of OTUs were identified by using blastn in UNITE: p, phylum; o, order; f, family; g, genus; s, species.

Table S6 Mantel test to determine the correlations between the environmental variables and bacterial community structures at eight successional stages.

| Variables | Wetland group | | Dry-land group | | All samples | |
| --- | --- | --- | --- | --- | --- | --- |
|  | r | p | r | p | r | p |
| pH | 0.051 | 0.305 | **0.429** | 0.004** | 0.059 | 0.140 |
| SOC | 0.086 | 0.213 | **0.471** | 0.001** | **0.121** | 0.046* |
| TN | **0.601** | 0.005** | 0.040 | 0.326 | **0.121** | 0.049* |
| AN | **0.647** | 0.004** | **0.538** | 0.005** | 0.098 | 0.063 |
| TP | 0.146 | 0.123 | **0.281** | 0.026* | 0.092 | 0.126 |
| AP | -0.062 | 0.656 | 0.198 | 0.074 | **0.306** | 0.004** |
| MC | **0.712** | 0.001* | 0.204 | 0.051 | **0.626** | 0.001** |

Wetland group indicated the original natural wetland (NW), wetland edge (EW), shrub-invaded wetland (IW), shrub-dominated wetland (DW).

Dry-land group indicated the young-*Betula* forest (YB), mature-*Betula* forest (MB), *Populus* and *Betula* mixed forest (PB), and conifer forest (CF).

Only significant correlations (**, P < 0.01; *, P < 0.05) were shown in bold number.

SOC: soil organic carbon; TN: total nitrogen; AN: available nitrogen; TP: total phosphorus; AP: available phosphorus; MC: moisture content.

Table S7 Mantel test to determine the correlations between the environmental variables and fungal community compositions at eight successional stages.

| Variables | Wetland group | | Dry-land group | | All samples | |
| --- | --- | --- | --- | --- | --- | --- |
|  | r | p | r | p | r | p |
| pH | -0.023 | 0.494 | **0.777** | 0.001** | **0.152** | 0.025* |
| SOC | 0.157 | 0.136 | **0.443** | 0.004** | 0.069 | 0.129 |
| TN | **0.575** | 0.004** | 0.156 | 0.110 | **0.167** | 0.021* |
| AN | **0.689** | 0.003** | 0.044 | 0.291 | **0.180** | 0.014** |
| TP | **0.243** | 0.043* | 0.229 | 0.062 | 0.120 | 0.069 |
| AP | -0.067 | 0.688 | **0.221** | 0.048* | **0.476** | 0.001** |
| MC | **0.693** | 0.001** | 0.001 | 0.508 | 0.657 | 0.201 |

Wetland group indicated the original natural wetland (NW), wetland edge (EW), shrub-invaded wetland (IW), shrub-dominated wetland (DW).

Dry-land group indicated the young-*Betula* forest (YB), mature-*Betula* forest (MB), *Populus* and *Betula* mixed forest (PB), and conifer forest (CF).

Only significant correlations (**, P < 0.01; *, P < 0.05) were shown in bold number.

SOC: soil organic carbon; TN: total nitrogen; AN: available nitrogen; TP: total phosphorus; AP: available phosphorus; MC: moisture content.
